# Trifunctional Epoxy Resin Composites Modified by Soluble Electrospun Veils: Effect on the Viscoelastic and Morphological Properties

**DOI:** 10.3390/ma11030405

**Published:** 2018-03-09

**Authors:** Giulia Ognibene, Salvatore Mannino, Maria Elena Fragalà, Gianluca Cicala

**Affiliations:** 1 Department of Civil Engineering and Architecture, University of Catania, Viale Andrea Doria 6, 95125 Catania, Italy; giuliaognibene@live.com (G.O.); salvatoremannino@hotmail.com (S.M.); 2 Italian Consortium for Materials Science and Technology Reseacch Unit Catania, Viale Andrea Doria 6, 95125 Catania, Italy; me.fragala@unict.it; 3 Department of Chemical Science, University of Catania, Viale Andrea Doria 6, 95125 Catania, Italy

**Keywords:** composites, toughening, electrospinning

## Abstract

Electrospun veils from copolyethersulfones (coPES) were prepared as soluble interlaminar veils for carbon fiber/epoxy composites. Neat, resin samples were impregnated into coPES veils with unmodified resin, while dry carbon fabrics were covered with electrospun veils and then infused with the unmodified epoxy resin to prepare reinforced laminates. The thermoplastic content varied from 10 wt% to 20 wt%. TGAP epoxy monomer showed improved and fast dissolution for all the temperatures tested. The unreinforced samples were cured first at 180 °C for 2 h and then were post-cured at 220 °C for 3 h. These sample showed a high dependence on the curing cycle. Carbon reinforced samples showed significant differences compared to the neat resin samples in terms of both viscoelastic and morphological properties.

## 1. Introduction

Epoxy resins are widely used as highly crosslinked materials in different fields ranging from civil to automotive and aerospace. Epoxy resins are the preferred choice in the aerospace field because of their high glass transition temperatures combined with their high stiffness and their solvent resistance [1,2,3,4,5]. However, the use of epoxy composites for primary aircraft structures was initially limited by the inherent brittleness of the unmodified epoxy resins. These limitations were overcome by the development of several toughening strategies. Among the different strategies identified, the use of engineering thermoplastics as toughening agents were the most successful. 

Cycom™ 977-2 and Hexcel 8552 are two examples of structural toughened prepregs, based on the addition of engineering thermoplastics. Both systems are qualified for use in primary aircraft structures. The two systems, after curing, present glass transition temperatures of 212 °C and 200 °C, respectively. However, most existing toughening strategies have been designed for prepreg processing only. Recently, the development of infusion-based techniques stressed the need for the development of an alternative toughening strategy that could avoid the increase of the resin viscosity found with thermoplastic blending. Cytec Engineered Materials addressed this need by developing the Priform™ technology that is based on the use of soluble melt extruded fibers based on their proprietary thermoplastic toughening agents [6]. This approach led to the development of materials manufactured by infusion but with the same properties of Cycom™ 977-2.

The use of soluble filaments dispersed in epoxy resins was proposed by using electrospun thermoplastic fibers as an alternative approach to the use of melted filaments or films. Several studied have been reported on the use of soluble electrospun fibers [7,8,9,10] but the effects of epoxy blend composition (i.e., epoxy monomer type and hardener type) on the fiber dissolution has not been analyzed thoroughly. Since different epoxy monomers and several types of hardeners are available, it is possible to produce epoxy formulations with different properties [11]. Therefore, the effect of formulation components, which include multifunctional epoxy monomers, is of high practical interest.

Multifunctional epoxies have higher crosslink densities than difunctional monomers [12,13] resulting in higher strengths, stiffness, and glass transition temperatures (Tg). T300/914, for example, is a commercial prepreg system consisting of carbon fibers embedded within a blend of tetraglycidyl 4,4-diaminodiphenyl-methane (TGDDM) and triglycidyl-p-aminophenol (TGAP) hardened with dicyandiamide (DICY) and/or diaminodiphenylsulfone (DDS), to which a small percentage of polyethersulfone (PES) is added [14]. Hourston et al. [15] reported the optimization of toughness for epoxy matrixes by mixing di-and tri-functional epoxide monomers. The effect on cure kinetics of a trifunctionalepoxy prepolymer TGAP on a thermoplastic-modified epoxy blend was reported by Bonnaud et al. [16]. The TGAP monomer increased the cure rate of the difunctional epoxy-based formulation. In a previous paper, the effect of the polymer molar mass on epoxy modified with electrospun veils obtained from copolyethersulfones was reported [17]. In a recent paper, we showed the effect of blending different ratios of TGAP and DGEBA on the properties of epoxy blends modified with copolyethersulfones [11]. 

The use of copolyethersufoneselectrospun veils with different molar mass was analyzed in a recent paper [17]. However, the study was limited to the use of DGEBA as epoxy monomer. In the present paper, we extend the study by using the trifunctional epoxy monomer triglycidyl-p-aminophenol (TGAP) in substitution of the difunctional diglycidylether of bisphenol A (DGEBA). 

## 2. Experimental

### 2.1. Materials

Theepoxy resin used was triglycidyl-p-aminophenol (TGAP) (Huntsman, Basel, Switzerland) with an equivalent weight of 101. The curing agent was 4,4′-methylene *bis*(2,6-diethylaniline) (MDEA) supplied by Lonza, Basel, Switzerland. The thermoplastic polymer was a copolyethersulfone, synthetized by the authors, with a number average molar mass of 9000 g/mol (coPES 9k) and bearing amino-phenol end groups. The details regarding the synthesis of coPES are reported elsewhere [18]. Plain carbon fabrics (C-200T from Prochima, Milan, Italy) with an areal weight of 200 gsm (grams per square meter) were used for the preparation of the reinforced samples.

### 2.2. Electrospun Veil Preparation

In the first step of coPES 9k veil production, 5.00 g of the polymer were dissolved in a solvent mixture (5.00 mL *N*,*N*-dimethylformamide (DMF) and 5.00 mL of Toluene) and stirred for 2 h at 40 °C. After the complete polymer dissolution, the solution was placed in a 3-mL medical syringe and electrospun at a flow rate of 60 µL/min, 21 kV ddp and a 10-cm needle-collector gap onto a rotating drum (200 rpm). The polymer fibers were spun directly onto the carbon fabric that was stuck on the rotating collector using conductive carbon tape. To obtain the desired wt% ratios of toughener in the composite, a selected gsmamount of coPES 9k veil was placed through the interlaminar regions according to the areal density of the carbon fabric.

### 2.3. Neat Resin Preparation

Cured neat resin samples were prepared to study the effect of the neat matrix without carbon fiber reinforcements. For this purpose, a selected amount of electrospun veils were laid up in the aluminum dish. In a second step the unmodified epoxy resin (preheated at 130 °C for 5 min) was poured on the veils to impregnate them. The dish was transferred to the oven, which was set at 130 °C and kept at this temperature for 30 min. The oven temperature was increased by 2 °C/min up to 180 °C and held at that value for 3 h. This cure rate was selected as it is the commonly used for aerospace qualified prepreg system. After this curing cycle, the samples were also post-cured at 220 °C for 3 h to fully develop their network. At the end of the curing cycle, the samples were left to cool down slowly at room temperature.

### 2.4. Composite Laminate Production

Six layers of dry carbon fabrics with electrospun veils were stacked on a steel plate. Around the perimeter of the layered stack, an adhesive silicone tape was placed to provide a suitable seal and a flexible vacuum bag was placed on top. An inlet tube and an outlet tube were put inside the vacuum bag. The inlet tube was connected by a valve to a pot filled with unmodified epoxy resin while the outlet tube was connected to a vacuum pump. The vacuum was applied while the inlet valve was closed to compact the layers and to remove excess air. The steel plate with all the stacked layers were placed in an oven preheated to 130 °C. The epoxy resin was vacuum infused into the stacked layers, which was maintained at 130 °C under a constant vacuum (75 cmHg). The temperature was kept at 130 °C for 30 min and then increased by 2 °C/min up to 180 °C and held at that value for 3 h. A similar cure cycle was used for the prepreg composites. However, in this case, the epoxy/coPES blends were used to impregnate the carbon fabrics which were then laid on the steel plate. The obtained stack was compacted using a vacuum bag at room temperature for 15 min. The stack was placed in an oven preheated to 130 °C for 30 min. The temperature was then increased to 180 °C and held at that value for 3 h. After this curing cycle the laminates were also post-cured at 220 °C for 3 h. At the end of the curing cycle, the samples were left to cool down slowly at room temperature.

## 3. Characterization

### 3.1. Hot-Stage Microscopy

Hot-stage microscopy was used to observe the dissolution of coPES fiber in an epoxy resin at different temperatures. A Linkam THMS 600 hot stage (The McCrone Group, Westmont, IN, USA) with TP-90 controller was fitted to an Olympus BX60 optical microscope (Milan, Italy). The unmodified epoxy resin was first preheated to the testing temperature. Next, a drop of resin was placed on a thin glass microscope slide where coPES fibers were spun before, after that another thin glass slide was placed on top of the veil. The prepared sample was subsequently held in the hot stage at the testing temperature and observed under the optical microscope, with the time needed to dissolve the veil being noted. This procedure was repeated for testing temperatures of 130, 120, 110, 100, 90, 80, 70 and 60 °C.

### 3.2. Dynamic Mechanical Analysis (DMA)

DMA of cured samples was carried out in single cantilever bending mode using a dynamic mechanical thermal analyzer (TRITEC by Triton Technology, Mansfield, MA, USA). All specimens were vacuum dried at 40 °C overnight before testing. The test was performed in accordance to ASTM E1640 by using samples of size 30 mm × 10 mm × 5 mm. All the cured samples were analyzed at a frequency of 1 Hz, with a 2 °C/min heating rate. The maxima on tanδ versus temperature curves were determined to identify the α-relaxations associated with the glass transitions. All samples were vacuum dried at 40 °C overnight before testing.

### 3.3. Scanning Electron Microscopy (SEM)

SEM micrographs were obtained with a SEM EVO-MA15 by Zeiss, Cambridge, UK. The electrospun veils, the cured matrixes and the cured laminates was analyzed. The cured neat matrixes were fractured in liquid nitrogen and then etched, using a mixture of sulfuric acid/distilled water (3:2) before sputter-coating. For the etching treatment, the samples were immersed in the acid mixture and stirred for 20 min. The acid mixture was used to etch the epoxy phase increasing the contrast between the thermoplastic and epoxy phases. The cured laminates were polished before etching. All etched samples were gold sputtered before the analysis without any other treatment.

## 4. Results and Discussion

The results on the neat resin samples are presented first, while the data for the composite laminates reinforced with carbon fibers are discussed after.

### 4.1. Neat Resin Samples

**Figure 1:**
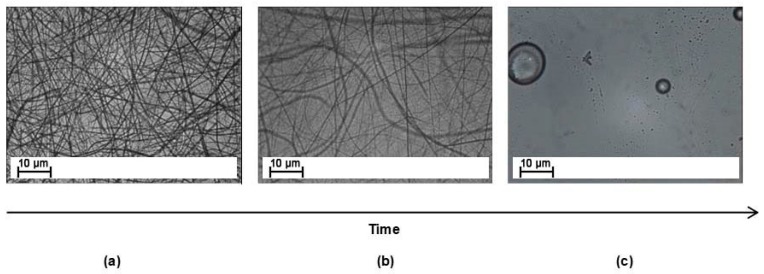
Hot-stage microscope screenshots for systems modified with coPES 9k treated at 100 °C at different times: (**a**) t = 0; (**b**) half dissolution; and (**c**) complete dissolution.

To evaluate the dissolution rate of the electrospun fibers at different temperatures, hot-stage microscopy was used. Figure 1 shows three representative screenshots of the experiment performed at 100 °C. Figure 1a shows the veil immersed in the epoxy resin at the start of the test. After few seconds, the electrospun fibers were no longer visible (Figure 1b), and, after 1 min, all fibers disappeared because the veil was fully dissolved in the resin. The time at which no fibers were visible was recorded as the dissolution time.

**Figure 2:**
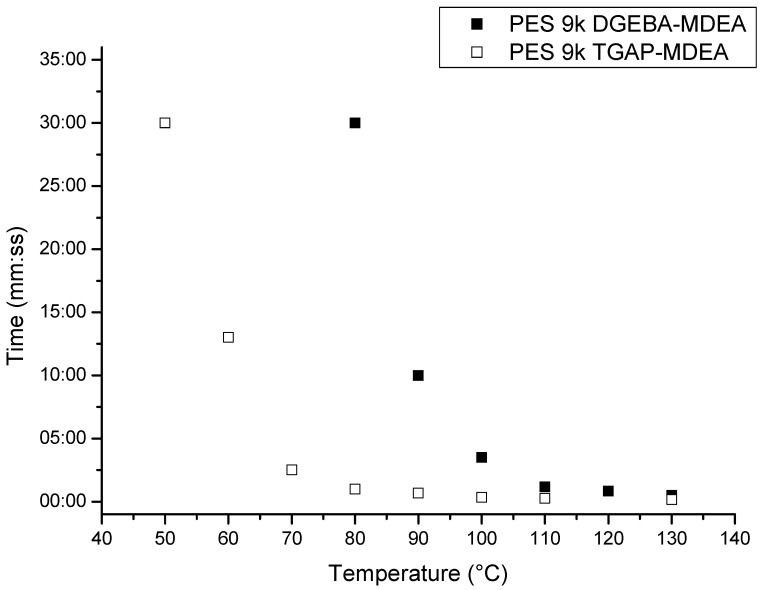
Dissolution time at different temperatures for coPES 9k in DGEBA-MDEA (data from [7]) and in TGAP-MDEA.

All the dissolution times recorded for different temperatures are plotted in Figure 2. The graph shows the comparison of the dissolution times at different temperatures for the veils of coPES 9k in two different epoxy resins cured by MDEA: TGAP and DGEBA. The data of the fibers dissolution in the DGEBA/MDEA system were obtained previously and they are reported here for comparison purposes [17]. The coPESveil showed similar dissolution times in the two resins between 100 °C and 130 °C. However, for temperatures lower than 100 °C, the veil showed faster dissolution for TGAP/MDEA than for DGEBA/MDEA. At 90 °C, the veil dissolved in 7 min with DGEBA/MDEA while less than 1 min was needed to observe full dissolution with TGAP/MDEA. Furthermore, at temperatures lower than 80 °C the complete dissolution of the veils was not observed for DGEBA/MDEA, while using TGAP/MDEA, veil’s dissolution still occurred. The veils showed full dissolution with TGAP/MDEA for temperatures down to 60 °C. These results confirmed that TGAP is a better solvent for coPES as it was already demonstrated in previous studies [11]. Others authors proved that TGAP increases the miscibility in epoxy/PES blends due to its higher compatibility with PES [16].

**Figure 3:**
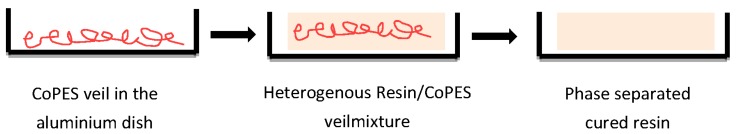
Schematics of soluble veil preparation route for neat resin samples.

To prepare the neat resin samples, the unmodified epoxy resin was poured in the aluminum dish where the electrospun veils were previously laid up (Figure 3). The impregnated veils were cured in an oven to study the veil dissolution. The coPES veil content varied from 10 wt% to 20 wt% in these samples. These polymer percentages were selected since they are usually reported in toughening studies of PES/Epoxy blends [6]. After 10 min at 130 °C, the samples became transparent with full dissolution of the veils. At the end of curing the samples became opaque. The samples obtained were cut for DMA and SEM investigation.

**Figure 4:**
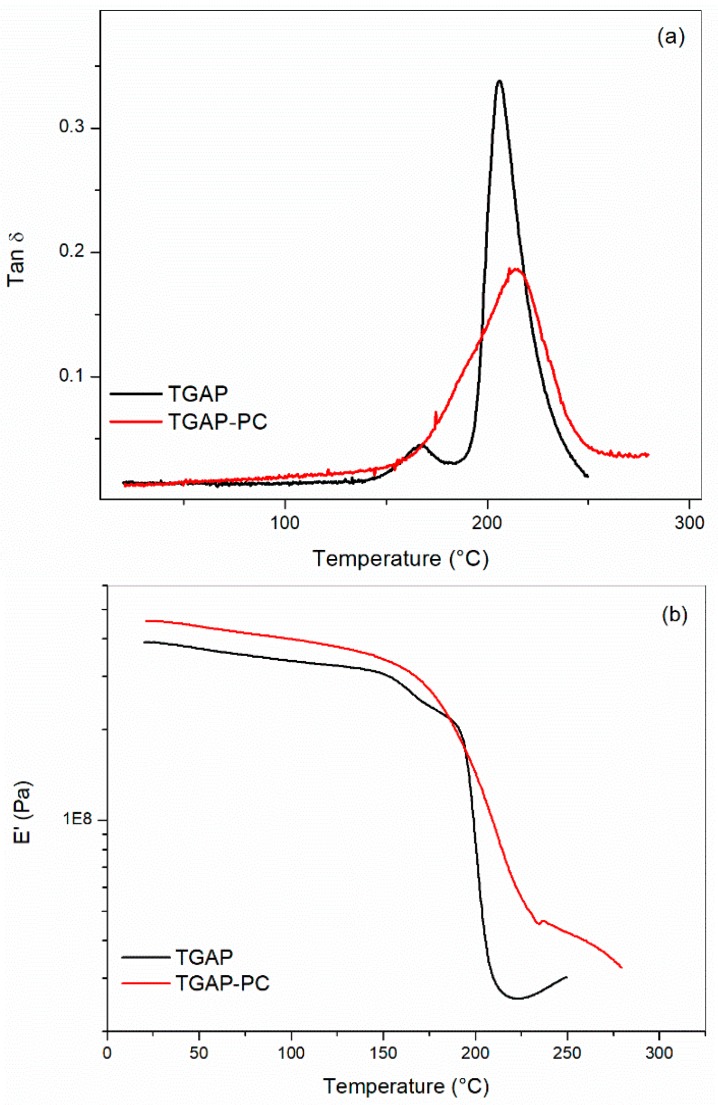
DMA comparison for the neat matrixes before (TGAP) and after post-curing (TGAP-PC): (**a**) tanδ versus temperature; and (**b**) Storage modulus (E’) versus temperature.

The unmodified TGAP/MDEA system showed one main tanδ peak centered at 206 °C and a small tanδ at 166 °C before the post-curing (Figure 4a). Two-step decrease in the storage modulus (E’) was observed for the system cured at 180 °C (Figure 4b). For this system, the storage modulus started to increase at about 215 °C during the DMA. Similar behavior was observed by Kim and Inoue for TGAP cured by 4,4′-diaminodiphenylsulfone (DDS) at 180 °C for different curing times [19]. In their systems, Kim and Inoue observed the disappearance of the low temperature peak upon increasing of the curing time. The low temperature peak was correlated to an epoxy oligomer phase which was converted to a crosslinked phase leading to a more homogenous epoxy network after higher curing times. Similarly, in our samples, the lower temperature peak disappeared upon post curing at 220 °C leading to the presence of one single wide peak centered at 214 °C (Figure 4a) and, for the storage modulus, only one step decrease was observed after post-curing (Figure 4b). However, it must be noted that, in contrast to what was reported by Kim and Inoue, in our samples, we found an increase of the high temperature peak instead of a decrease upon post-curing. This difference could be interpreted as an improved conversion of the unreacted epoxy groups leading to higher Tg because post-curing was carried out at higher temperature (i.e., 220 °C) than for the first curing step (i.e., 180 °C).

**Figure 5:**
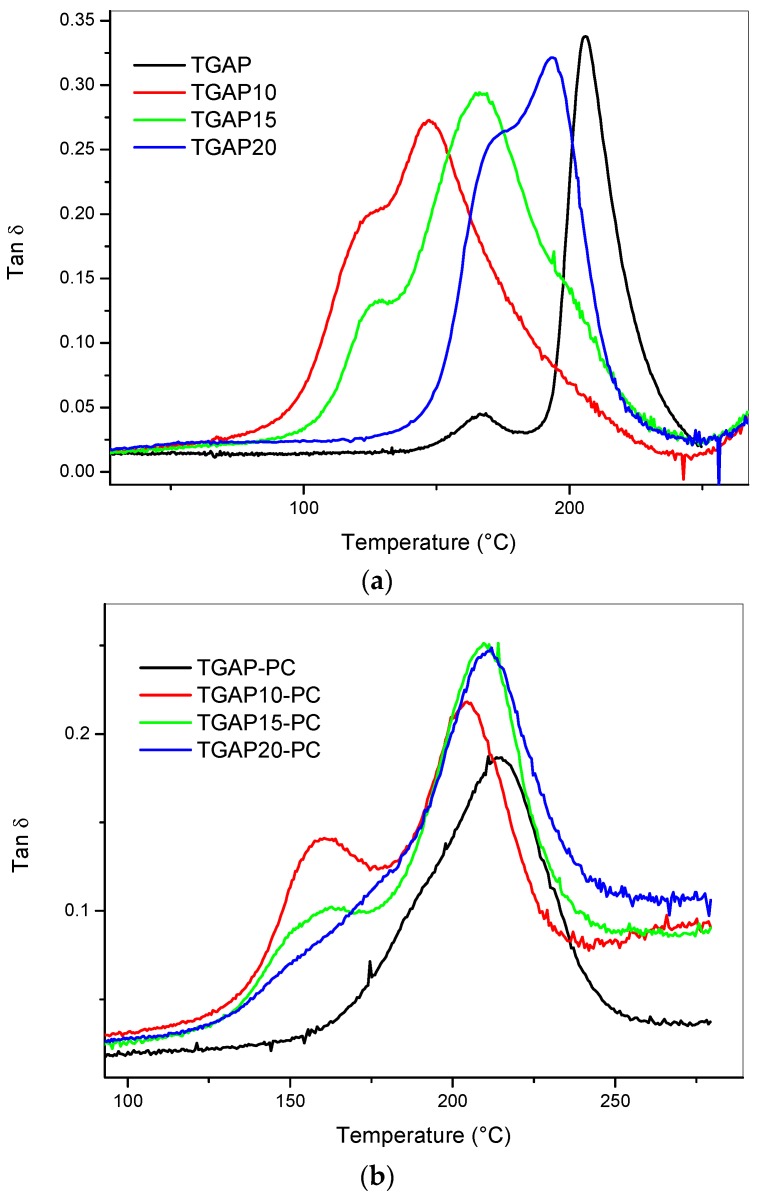
Tanδ versus temperature curves for the neat samples with different veil contents: after curing (**a**); and post-curing (**b**).

The tanδ versus temperature behavior for the blends obtained by the impregnation of the coPESelectrospun veil is reported in Figure 5. The modified blends after the first curing step showed a main relaxation peak and a shoulder at lower temperatures (Figure 5a). The peaks for each coPES composition appeared broader than for the unmodified resins. When increasing the coPES content, the peaks shifted to lower temperatures compared to the unmodified TGAP/MDEA. The tanδ behavior changed after post-curing at 220 °C (Figure 5b) showing a main peak centered between 205 °C and 212 °C and a smaller peak centered between 158 °C and 161 °C. 

Similar results were confirmed analyzing the storage modulus curves (see Supplementary Materials, Figure S1a,b). The storage modulus curves showed higher modulus in the glass region for the modified systems compared to the unmodified resins. This can be explained as the results, for the modified blends, of the reinforcing effect of the stiff thermoplastic domains in these systems. For temperatures above 205°C, which corresponds to the Tg of pure coPES [17], the behavior was different with the modified blends displaying lower modulus than the unmodified blends.

Varley et al. [20] reported three tanδ peaks after curing at 150 °C for 16 h for blends based on TGAP/DDS modified with polysulfone (PSF). The tanδ versus temperature turned to two-peak behavior after post-curing at 205 °C for 2 h. The change upon post-curing was explained as the results of the conversion of the unreacted epoxy species which was favored for systems displaying particulate morphologies. In addition, post-curing imparting greater mobility to all the species allowed further phase separation to occur resulting in the shift of the tanδ peak. 

The systems studied in the present paper presented relevant differences compared to the blends analyzed by Varley et al. [20]. First, the curing agent used was the MDEA in place of the DDS. MDEA is known to decrease the miscibility window for epoxy/PES blends [16]. Second, the amino-phenol end groups of the coPES used here allowed the co-reaction and an improved compatibility of coPES with the epoxy monomers as proved by several authors [21,22]. The use of MDEA led to phase separation in the early stage of curing, which due to the increased compatibility of our coPES with the epoxy monomers, presented a high amount of epoxy-curing species dispersed in the thermoplastic rich domains. Van Overbeke et al. [21] predicted, for similar blends cured at 160 °C, a content of 50% of epoxy-curing agent species remaining trapped in the thermoplastic-rich phase. In addition, the lack of affinity between MDEA and the coPES could further decrease the MDEA content in the coPES-rich phase resulting in stoichiometric imbalance with detrimental effect on epoxide conversion [23]. The presence of a high amount of not fully cured oligomer epoxy-curing agent species can lead to the decrease of the glass transition temperature of the thermoplastic rich phase. This decrease, occurring after the first cure step, was demonstrated by the low temperature peak occurring at temperatures (122–150 °C) lower than the Tg (205 °C, [17]) of the pure coPES.

Upon post-curing, the increased mobility of the coPES-rich phase allowed epoxy-curing agent species to demix from the thermoplastic phase purifying it and completing their conversion resulting in the formation of a higher crosslinked epoxy network with thermoplastic-rich phase dispersed. The tanδ curve versus temperature reported here showed distinct behavior compared to the blends reported previously [11]. However, it must be noted that the samples prepared in this paper underwent a more complex cure profile, needed to fully dissolve the electrospun veils, while, in the previous paper, the coPES powder was pre-dissolved in the epoxy monomer before adding MDEA to the blend. 

**Figure 6:**
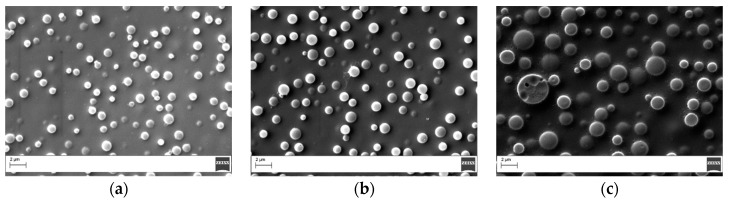
SEM images of the post-cured samples (magnification 10,000×): (**a**) 10 wt%; (**b**) 15 wt%; and (**c**) 20 wt%.

**Figure 7:**
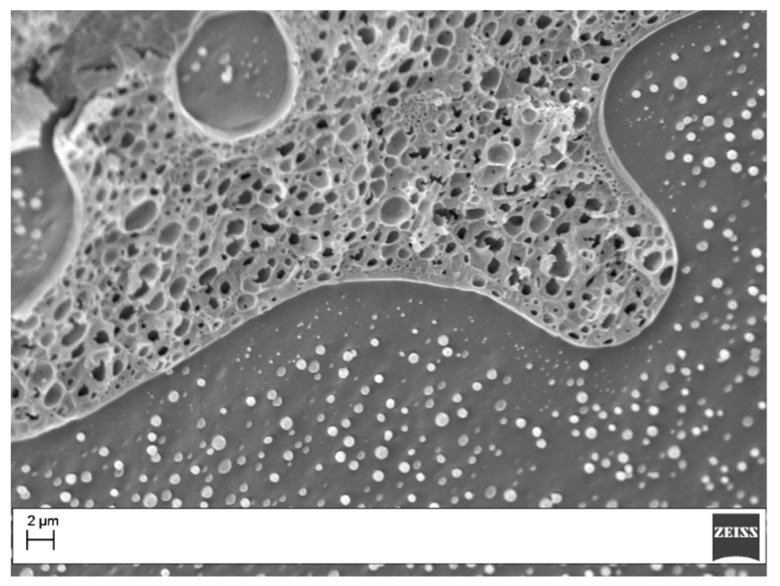
SEM image of the post-cured sample modified with 20 wt% of coPES (magnification 5000×).

Morphological analysis was carried out on both sample before and after post curing. The results for post-cured samples are reported in Figure 6. The analysis revealed for all the samples the presence of a predominant particulate morphology. The sample with 10 wt% showed particles with diameters ranging 0.5–1 µm, while, for the sample at 15 wt%, the particles diameter varied within 1–2 µm. The sample with 20 wt% of coPES showed bigger particles (1.2–3 µm) and some zone where phase inversion occurred (Figure 7). SEM analysis confirmed the full dissolution of the electrospun veils. The morphological analysis for the samples at 10 wt% and 15 wt% of coPES confirmed the DMA trend showing two tanδ peaks due to the coPES-rich (i.e., the particles) and epoxy-rich phase. The morphological analysis of the sample with 20 wt%, with the appearance of phase inverted regions, confirmed the DMA behavior of this sample too, which showed a wider tanδ peak due to the superposition of the two phases. 

### 4.2. Composite Laminate Samples

Carbon fiber reinforced laminates were produced by infusion to validate the veil dissolution in composite processing. For this purpose, hybrid carbon fabrics, with electrospun fibers deposited onto their surfaces, were infused by the unmodified TGAP/MDEA resin. The amount of the veil deposited was balanced against the areal weight of the carbon fabric to obtain the desired amount of the thermoplastic in the interlaminar region of the composite. The co-PES content varied, as for the neat resin samples, between 10 wt% and 20 wt%.

**Figure 8:**
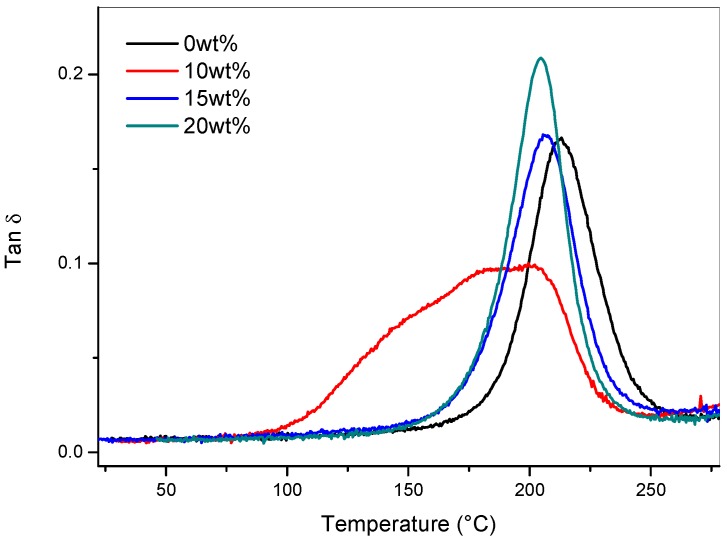
Tanδ versus temperature curves for the laminate with different veil contents after post-curing.

The samples after post-cured were analyzed by DMA (Figure 8). The laminates containing 15 wt% and 20 wt% of coPES veils showed a sharp tanδ peak centered at 206 °C and 204 °C, respectively. The laminate modified by 10 wt% displayed a wide tanδcentered at 191 °C. This peak was much wider than the peaks observed for the other two modified systems and its height was lower. The results of the DMA analysis (Figure 8) showed a different trend compared to the neat samples containing the same veil percentages (Figure 5b). 

**Figure 9:**
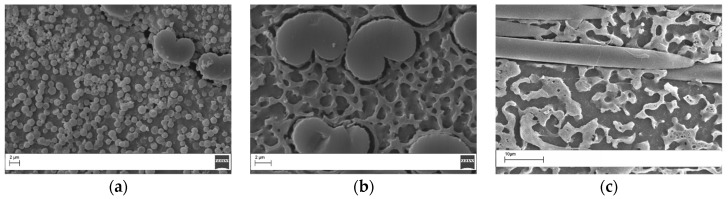
The interlaminar regions of the laminates modified with co-PES veils after post-curing: (**a**,**b**) magnification 10,000×; and (**c**) magnification 5000×.

The morphological analysis of the cryo-fractured laminates confirmed the findings of the DMA analysis. Interlaminar morphologies markedly different than for the neat resins were observed (Figure 9). Thermoplastic-rich particles denser and bigger than those observed for the neat resin sample (1.3–2 µm) for the laminates containing 10 wt% of coPES were found (0.5–1 µm, Figure 6a). The morphology for the laminates with 15 wt% and 20 wt% of coPES were co-continuous, while, for the analogous neat resin samples, particulate morphologies with some areas displaying phase inversion for the 20 wt% neat resin sample were displayed.

The presence of the reinforcing fibers had a pronounced effect on the final properties of the composites. Several causes can explain this result: the presence of the carbon fibers in the laminate acted as nucleating agent and hindered thermoplastic diffusion across the sample [24]; and the carbon fiber surface promoted stoichiometric gradients affecting the phase separation behavior [25]. Varley and Hodgking [24] studied and explained the different morphological behaviors in terms of the improved nucleation and agglomeration effect experience by the fiber surfaces. Similar effects seem to have occurred in the specimens analyzed here.

## 5. Conclusions

Viscoelastic and morphological properties of neat resins and fiber reinforced laminates modified with soluble coPES veils have been analyzed. The effect of different content of coPES veils and of different curing cycle was studied. 

The full dissolution of the coPES veil occurred after few minutes at the processing temperatures of interest because of the high solubility of coPES in the TGAP. This result is interesting for further developments of the soluble veil approach because TGAP can be envisaged as a method to tailor the properties of complex epoxy blend when fiber dissolution is a requirement. TGAP is an interesting epoxy monomer in this regard, as it allowed increasing Tg over difunctional epoxy monomers [11].

The use of the multifunctional epoxy monomer TGAP resulted in a complex viscoelastic behavior after the first curing step at 180 °C for the unmodified blend which showed two tanδ peaks in the DMA trace. The additional post-curing at 220 °C led to a single tanδpeak which can be ascribed to the formation of a more homogenous cured network. The modified blends with different coPES veil content showed even a more complex behavior because coPES veil, after its dissolution, undergone under phase separation with the formation of different morphologies. Neat resin samples showed multiple relaxation after the first curing step that shifted after post-curing because of demixing from thermoplastic-rich phase upon curing. This finding suggests that final properties of TGAP, over its advantages, are strongly dependent upon curing conditions. Similarly, when the resin was infused into carbon fabric hybridized with coPES veils the final properties of the system changed compared to neat resins. These results confirmed TGAP as a monomer with potential benefits for improving the final properties of soluble veil modified composites but, at the same time, as a monomer that should be employed carefully in formulation design. 

## Supplementary Materials

The following are available online at http://www.mdpi.com/1996-1944/11/3/405/s1, Figure S1: Storage modulus versus temperature for the neat samples with different veil contents after curing (a) and post-curing (b).
