# Supplementary material for: Trifunctional Epoxy Resin Composites Modified by Soluble Electrospun Veils: Effect on the Viscoelastic and Morphological Properties
Source: Materials (Basel). 2018 Mar 9;11(3):405. doi: 10.3390/ma11030405 (PMC5872984; doi:10.3390/ma11030405)
Supplement: Supplementary file 1 [file materials-11-00405-s001.docx]

**Supplementary Material**








**Figure S1** Storage modulus versus temperature for the neat samples with different veil contents after curing (**a**) and post-curing (**b**)
